# Translation and Cultural Adaptation of the Environmental Protectors Against Hospital Work Stress (ENPROS) Scale to Brazilian Portuguese

**DOI:** 10.3390/healthcare12222302

**Published:** 2024-11-18

**Authors:** Silmara Meneguin, Paula Astudillo Díaz, Ximena Osorio-Spuler, José Fausto de Morais, Camila Fernandes Pollo, Cesar de Oliveira, Juliana Pierami de Freitas

**Affiliations:** 1 Department of Nursing, Botucatu Medical School, São Paulo State University (UNESP), Botucatu 18618-687, SP, Brazil; camilapollo@hotmail.com (C.F.P.); juliana.pierami@unesp.br (J.P.d.F.); 2 Department of Nursing, Universidad de la Frontera, Temuco 4811230, Chile; paula.astudillo@ufrontera.cl (P.A.D.); ximena.osorio@ufrontera.cl (X.O.-S.); 3 Institute of Mathematics and Statistics, Federal University of Uberlândia, Uberlândia 38405-320, MG, Brazil; jfmorais.ufu@hotmail.com; 4 Department of Epidemiology & Public Health, University College London, London WC1E 6BT, UK; c.oliveira@ucl.ac.uk

**Keywords:** occupational health, health personnel, occupational stress, validation study

## Abstract

**Background:** Occupational stress and workplace violence are highly prevalent risk factors among healthcare professionals and can affect not only the psychosocial well-being of workers but also that of patients and healthcare organizations. **Objective:** The objective of this study is to translate and cross-culturally adapt the ENvironmental PRotectors against hOspital work Stress scale to facilitate future psychometric validation of the instrument. **Methods:** A methodological study was conducted at the School of Medicine of São Paulo State University (UNESP) in São Paulo, Brazil. This study involved three steps: translation and backtranslation by independent native language speakers, analysis by an expert panel, and a pre-test phase. Ten specialists adjusted and approved the final version for semantic, idiomatic, and cultural accuracy across nine items. **Results:** The content validity index was satisfactory (CVI ≥ 0.80). The final version was administered to 36 nursing and medical staff at a public hospital in São Paulo. No items were excluded from the instrument. Satisfactory content and face validity were achieved, and the criteria recommended by the literature were met. **Conclusions:** The Portuguese version of ENPROS is appropriate and culturally adapted for use in Brazil.

## 1. Introduction

Numerous researchers in recent decades have described the contemporary workplace environment as a substantial threat to safety and health, making workers vulnerable to occupational stress and illness [1]. Occupational stress is considered one of the leading occupational health problems that affects thousands of workers throughout the world [2,3]. The National Institute for Occupational Safety and Health (NIOSH) defines occupational stress as a set of harmful physical and emotional responses that occur when work requirements do not correspond to workers’ capacity, resources and needs [4].

In Brazil, occupational stress in health professionals has been linked to the high levels of physical and psychosocial demands associated with occupational characteristics and health conditions, which contribute to physical and emotional exhaustion, social isolation, and burnout among professionals, despite current labor legislation attempting to curb cases of workplace violence [5,6,7].

However, this work environment, often characterized by long hours, excessive productivity pressures, and a lack of work–life balance, can affect health, well-being, work productivity, and workplace safety [8]. Studies addressing occupational stress in a diverse group of workers [9,10], such as nurses, physicians, therapists, and others in different fields, showed that joint problems, such as long hours of activity, excessive work, inadequate human resources, emotional demands, administrative burden, and physical risks related to the work environment, are predictors of job stress [11,12,13].

Chronic exposure to these stressors has adverse effects on physical and mental health [14,15], predisposing workers to morbidities such as hypertension, musculoskeletal disorders, cardiovascular disease and substance abuse [16,17]. Workers with job stress have an estimated 50% higher risk of developing coronary artery disease [18]. Work stress is also one of the main reasons for lateness, absenteeism, and worker turnover [19], as well as reductions in organizational commitment, job satisfaction, care quality and organizational productivity [20,21].

Healthcare providers constitute a particularly vulnerable group, working in stressful environments and under considerable pressure that contributes to burnout. The magnitude of occupational stress in this group ranges from 27% to 87.4% [22,23,24]. Burnout in this population related to long-term work can lead to behavioral and psychiatric disorders, as well as poor quality of life [25,26].

Because occupational stress is a multivariate and slowly progressive [27] process in the health field, it is dangerous, and its management is essential to workers and society as a whole, as the quality of the care offered depends on the emotional state of these healthcare providers [27]. However, no documented strategies seek to mitigate the impact of job stress on the risk of becoming ill in Brazil [28]. Creating a secure and healthy workplace that prioritizes the preservation and enhancement of physical and mental health could bring about a new perspective in the field of health sciences. This approach would enable workers, organizations, and society to achieve optimal well-being [29,30].

Workplace violence against healthcare professionals is a critical issue that, unfortunately, has not yet received the necessary in-depth analysis. This phenomenon, which includes physical, verbal, and psychological aggression, occurs at an alarming rate in healthcare settings, affecting not only the mental and physical health of professionals but also the quality of care provided to patients. This problem is especially relevant in some areas, such as psychiatric wards, where the incidence of violence is higher, requiring specific prevention and support strategies to ensure the safety and well-being of staff [31].

A study that evaluated the characteristics of workplace violence in a psychiatric intensive care unit showed the need for protocols to address stressful workplace situations and adequate training for staff to deal with them. In addition, it suggests that an efficient reporting system is essential to monitor the frequency and severity of such incidents and assist in implementing more effective preventive measures to improve the work environment [32].

To focus on stress protectors, in 2005, researchers in Chile developed an instrument to measure job stress coping measures used by healthcare providers. This instrument was denominated ENvironmental PRotectors against hOspital work Stress (ENPROS), which has 40 items distributed among five dimensions and was developed from a qualitative study based on the grounded theory method. In 2017, ENPROS was administered to high-complexity hospitals in the Araucanía region of the country. Validity analysis revealed acceptable individual item reliability and factor loadings [33].

Given the lack of Brazilian studies producing or using scales in this context, the translation and cultural adaptation of ENPROS could provide a reliable, reproducible scale for data collection and analysis related to environmental protectors against job stress in the hospital environment. These measures could directly impact the health of these workers, their adequate coping with stress, and the quality of care offered to patients.

Therefore, the present study aimed to translate and cross-culturally adapt the ENvironmental PRotectors against hOspital work Stress scale to enable future psychometric validation of the instrument.

## 2. Methods

### Study Design and Development

Following international guidelines, a methodological study was conducted from July 2023 to January 2024 involving the translation and cross-cultural adaptation of ENPROS into Brazilian Portuguese [34].

1. Translation was carried out by two bilingual (Portuguese and Spanish) Brazilian translators without prior knowledge of the questionnaire. The translated versions were denominated T1 and T2 and were combined to obtain the T3 version employing components of T1 and T2. Using two translators allows for comparing and identifying possible discrepancies or differences in interpreting the original content, ensuring that specific nuances and meanings are preserved. This approach increases the validity of the translated instrument, as each translation can reveal alternative interpretations and adjustments needed to maintain fidelity to the original language, contributing to a more reliable and culturally adapted translation [35].
2. Backtranslation is a step necessary to determine whether the translated version reflects the same content as the original and address any inconsistencies. For this, the T3 version was backtranslated into Spanish by two bilingual, native Spanish-speaking translators who had no prior knowledge of the questionnaire. The result was two backtranslated versions, BT1 and BT2, which were subsequently combined into a single version, BT3.
3. Committee of Experts: The expert panel assessed the final translated version to obtain cross-cultural equivalence. The adapted Fehring criteria were used to select the experts on the committee. According to this author, to be considered an expert in a specialization, an individual must have a doctorate or master’s degree with dissertations or theses relevant to the topic of interest, published articles, and at least one year of clinical experience [36].

The panel members were selected based on their scientific knowledge from a national academic database, denominated the Lattes Platform, which has all curricular data on researchers, their research groups, and the fields in which they work for all higher education institutions in Brazil. The panel members were also part of a research group denominated Health Metrics, coordinated by one of the researchers. An invitation was sent via e-mail to each of the specialists selected, containing the terms of the agreement, a copy of the translated instrument (T3 version), and a form describing this study’s objective and instructions on performing the analysis/assessment.

The experts used a Likert scale ranging from 1 to 4 points to assess the instrument with regard to semantic, idiomatic, and cultural representativeness: 1 (item not representative), 2 (item needs significant revision to be representative), 3 (item needs minor revision to be representative) and 4 (item relevant or representative). The content validity index (CVI) was calculated for each item considering the sum scores of 3 and 4 divided by the total number of answers. A minimum CVI of 0.80 was established for an item to be considered adequate [37,38].

The expert panel was given 20 days to conclude the preliminary analysis. After receiving feedback, a synthesis version of the questionnaire was created based on the panel members’ recommendations. Expert participation is essential for several reasons, including technical competence, diversity of perspectives, and cultural relevance. Experts’ recommendations and validations can facilitate the implementation of the instrument and give the process greater credibility [39].

4. Pre-test phase: In this step, 36 healthcare providers were invited to answer the pre-final version of the translated instrument [34]. The objective of semantic analysis in this study was to assess whether all items were appropriate and understandable for the population for which the instrument was developed and should be administered to 30 to 40 individuals [34]. At this stage, randomly selected health professionals (physicians and nursing staff) working in various hospital units who consented to participate were included.

**Figure 1:**
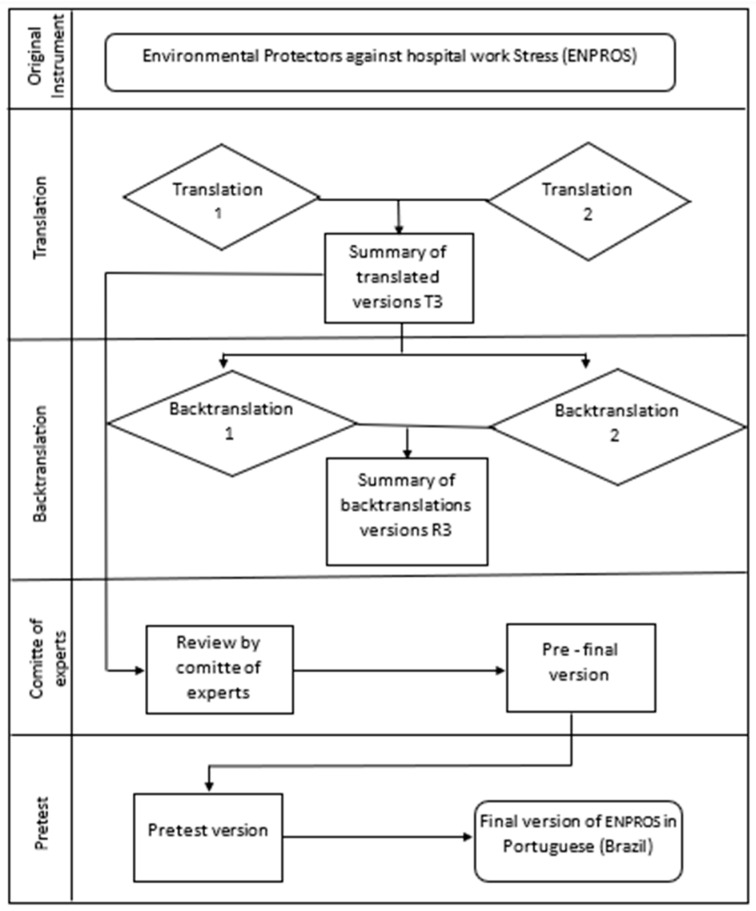
Flowchart of translation and cultural adaptation of ENPROS to Brazilian Portuguese, Botucatu, SP, Brazil, 2024.

Each item was assessed for adequacy/understanding: “Yes”, “No”, or “Partially”. The items were validated after achieving a minimum agreement level of 75% of affirmative answers. The items with lower agreement were considered to require alteration [40]. Figure 1 displays the flowchart of the translation and cultural adaptation process. After the expert panel analyzed the instrument and executed the pretest, the translated instrument was sent to the authors of the original scale for consolidation into the final version.

This study was authorized by the original instrument authors and approved by the institutional Human Research Ethics Committee on 10 October 2023 (certificate number 2.772.325). Informed consent was obtained from all subjects involved in this study. This study was reported following the *Standards for Quality Improvement Reporting Excellence* (SQUIRE 2.0) [41].

## 3. Results

The translation and cultural adaptation of ENPROS to the Portuguese language spoken in Brazil were carried out according to the method proposed in this research. The summary was obtained in a single version called T3, which was then subjected to the backtranslation process into Spanish, and the summary was obtained in a single version called R3.

A Committee of Experts evaluated Version T3 to determine cross-cultural equivalence. Ten judges were chosen for convenience through non-probabilistic sampling by analyzing their curriculum and scientific knowledge.

All ten judges were women (100%). Eight (80%) were nurses and two (20%) were physicians. Six (60%) panel members worked in clinical care settings. Five (50%) worked in the field between 11 and 15 years. Six (60%) members had doctoral degrees, and seven (70%) had experience with scale development and/or cultural adaptation. Four (40%) members had graduated 11 to 15 years before the present study, and the other four (40%) had graduated more than 20 years before this study.

The expert panel made changes to two section statements and nine items (Items 2, 10, 26, 29, 30, 34, 36, 37 and 40). The main changes regarded the need for agreement between subject and verb, using synonyms and rewriting of the item to ensure better understanding. These and other items that were altered by the expert panel are in bold type in Table 1.

Each member of the expert panel assessed each of the 40 ENPROS items and each of the nine items directed at sociodemographic characterization. The mean CVI was as follows: semantic = 0.94; idiomatic = 0.94; cultural = 0.97. The global mean CVI considering all categories was 0.95 and considered substantial, as the literature recommends a minimum CVI ≥ 0.80 to be considered acceptable. The results demonstrate that the items that compose ENPROS translated and adapted to Brazilian Portuguese have adequate representativity. Moreover, 45% and 72.5% of the translated and adapted items had CVIs of 1.0 in the semantic and idiomatic categories, respectively. In the cultural category, 52.5% had CVIs of 9.0, whereas all other items had CVIs ≥ 8.0. The items that did not obtain the maximum CVI had values of 0.80.

The pretest version was administered to 36 health professionals at a tertiary public hospital. The mean age was 37.2 ± 10.8 years. Most participants were women (81.3%) and nursing staff (61.3%) and had between 5 and 15 years of experience (52.8%). Fifteen (41.7%) had a higher education, eleven (30.5%) had a specialty and only one (2.8%) had a doctoral degree. Concerning family income, 14 (38.1%) reported between BRL 3100 and BRL 5000 per month and 13 (36.1%) reported between BRL 5100 and BRL 10,000 per month. The average time required to answer the questionnaire was 19 ± 13 min, and the rate of agreement on the assessment was 97%.

ENPROS, translated and adapted to Brazilian Portuguese, remained substantially identical to the original version, as the scale did not undergo significant changes or deviations compared to the original version.

## 4. Discussion

The present study used the systematic method described in the literature to translate, culturally adapt, and validate ENPROS’s content into Brazilian Portuguese. This study emerged from the intention to import international scientific knowledge in the field to enable investigations in health. Formal, objective instruments for data collection in scientific studies in diverse fields of knowledge are scarce in Brazil, especially concerning protective factors against work-related stress in health professionals. Thus, the use of international instruments has become increasingly frequent, with a strong trend of methodological studies involving translation and cultural adaptation in the country [42].

Cross-cultural validation confirms that the original instrument and translated version are comparable [43], with the measure equivalent and precise for use in Brazil. The literature describes this step as critical for health professionals’ inferences, as it ensures their understanding of the instrument [44].

With regard to the changes proposed by the expert panel, two were made to statements corresponding to two different sections: Section I, to which a change was made only to the writing format, and Section V. The committee also made changes in nine items, with single words being replaced with synonyms and words for adequate cultural understanding being included. For the translated version of Item 40, the expert panel suggested changing the wording and deleting some words. According to the qualitative study conducted in Chile, which originated the central topics for the creation of the instrument, the ‘teamwork’ construct is founded on the assumption of a sense of belonging and the psychosocial environment of interpersonal relationships. Thus, the structural change made to the item—with the exclusion of some words—did not affect the main message. On the contrary, the change resulted in a better understanding of the item, highlighting the main idea of a sense of belonging and the psychosocial environment of interpersonal relationships by discussing the concept of teamwork despite having conflicts and divergences in the family or group in question.

An assessment tool’s translation and cultural adaptation encompasses multiple aspects and may result in adaptations to the items [45]. However, none of the items on the ENPROS were excluded after translation because their CVI was lower than 0.80 [46], demonstrating that the translated version is compatible with the original scale.

The participants, who predominantly performed care functions at tertiary-level hospitals, adhered closely to the pretest administration. The original instrument was also highly compatible, suggesting a successful translation and adaptation process, considering the participants’ understanding of the scale. This information is relevant to future analyses of the proposed instrument’s use.

The newly translated and culturally adapted version of the ENPROS will allow for an accurate assessment of the experiences of Brazilian workers, considering the cultural and social particularities that may influence the perception of occupational stress. This will provide valuable data for researchers and health professionals, allowing for the identification of critical areas that require specific interventions. Furthermore, using the ENPROS scale can help foster discussions on mental health in the workplace, highlighting the importance of worker well-being in a context where occupational health is often neglected. By better understanding the sources of stress and its effects, organizations can develop more effective policies and practices to support the mental health of their employees.

In this context, conducting a construct validation study of the ENPROS scale will be essential to ensure that the instrument effectively measures occupational stress in specific contexts, such as Brazil. This future validation will not only ensure that the scale adequately captures the different protective aspects against stress in the workplace but will also provide robust evidence of its reliability and validity.

Hopefully, this instrument will be helpful within the Brazilian healthcare system, which is facing significant pressure due to a combination of structural, financial and operational factors, such as system overload, shortage of professionals, insufficient funding and the increase in chronic diseases resulting from population aging. These factors make the healthcare system environment in Brazil challenging, requiring structural reforms and new investments to balance the system’s capacity with the population’s growing demands [47,48,49,50].

In this scenario, where mental health and occupational stress are increasingly in the spotlight, validating the ENPROS scale becomes a crucial tool for promoting healthier and more productive work environments. It allows organizations to recognize the importance of mental healthcare and adopt concrete measures to mitigate occupational stress among their professionals.

## 5. Limitations

The main limitation of this work was the lack of other versions of ENPROS published in different languages to compare the results. Another possible limitation is the lack of a pilot study, although the literature adopted [40] does not require one.

## 6. Conclusions

The Portuguese version of the ENvironmental PRotectors against hOspital work Stress (ENPROS) showed good linguistics and content validity, revealing its potential for use in hospital practice and future research. To conclude its validation process, the psychometric assessment of its properties is necessary, using the Classical Test Theory.

Considering the scarcity of studies involving job stress coping strategies, the translation and cultural adaptation of this instrument into Brazilian Portuguese will enable health services to reconsider their current approaches, resulting in improvements to the health of workers. This instrument will foster further research on work stress and assessing working conditions among healthcare providers.

## Figures and Tables

**Table 1:** Items on the translated version of ENPROS with CVI = 0.80 and/or rewritten by the expert panel (changes in bold type), Botucatu, SP, Brazil, 2024.

Item	Version	Text	CVI *		
			Semantic	Idiomatic	Cultural
Section I					
Statement	Original	*Respecto a la organización, ¿qué importancia tienen para usted las siguientes afirmaciones?* [With regards to the organization, what importance do the following statements have for you?]			
	Translated	What is the importance to you of the following statements with regards to the institution? Mark or circle your choice.			
	Expert panel version	With regards to the institutional organization, which of the following statement have importance to you?			
2	Original	*La organización debería dar respaldo legal a sus trabajadores ante situaciones conflictivas*. [The organization should give legal backing to its workers in conflicting situations.]			
	Translated	The institution should offer juridical support to its employees in situations of conflict.			
	Expert panel version	The institution should offer juridical backing to its employees in situations of conflict.	1.000	0.900	1.000
Section II					
10	Original	*En cada puesto de trabajo debería darse a conocer los elementos que se toman en cuenta para evaluar el desempeño.* [At every work post, the elements that are considered to evaluate performance should be known.]			
	Translated	The professional performance evaluation criteria for each position should be known.			
	Expert panel version	The professional performance evaluation criteria for each position should be known disclosed.	0.900	0.800	1.000
Section IV					
26	Original	*Debería existir un espacio que permita tener privacidad*. [There should be a space that enables having privacy.]			
	Translated	There should be [word in Portuguese: *haver*] a space that enables having privacy.			
	Expert panel version	There should be [synonym in Portuguese: *ter*] a space that enables having privacy	1.000	0.900	1.000
Section V					
Statement	Original	*Respecto al trabajo en equipo ambiente psicosocial: ¿qué importancia tienen para usted las siguientes afirmaciones? Marque con una cruz o un círculo su opción. (Llamamos ambiente psicosocial a las relaciones de las personas entre sí, y las relaciones de las personas y su ambiente social al interior de la unidad de trabajo).* [With regards to teamwork/psychosocial environment, what importance do the following statements have for you? Mark your answer with an x or circle. (We call psychosocial environment the relationships among people and relationships between people and their social environment within the work unit).			
	Translated	With regards to teamwork/psychosocial environment, what importance do the following statements have for you? (Psychosocial environment means relationships among people).			
	Expert panel version	With regards to teamwork/psychosocial environment, what importance do the following statements have for you?			
29	Original	*Cada miembro del equipo debería estar comprometido en cumplir los objetivos de la organización.* [Each member of the team should be committed to meeting the goals of the organization.]			
	Translated	Each member of the team should be engaged in achieving the goals of the organization.			
	Expert panel version	Each member of the team should be engaged in achieving the goals of the institution.	1.000	0.900	1.000
30	Original	*Cada miembro del equipo que asista a una capacitación o congreso, debería compartir el conocimiento adquirido.* [Each member of the team who attends a workshop or conference should share the acquired knowledge.]			
	Translated	Each member of the team who participates in training or a conference should share the acquired knowledge.			
	Expert panel version	Each member of the team who participates in a training course or conference should share the acquired knowledge.	1.000	0.900	1.000
34	Original	*Debería existir un lenguaje sin gritos ni estridencias dentro del equipo.* [There should be a language without shouting or stridency within the team.]			
	Translated	There should be verbal communication without shouting or raised voices.			
	Expert panel version	Communication among the team should be without shouting or strident noises.	0.900	0.800	0.900
36	Original	*Debería existir un ambiente de trabajo cordial, afectivo y amigable.* {There should be a cordial, affective, friendly work environment.]			
	Translated	There should be a cordial, affectionate, friendly work environment.			
	Expert panel version	There should be a pleasant, affectionate, good-humored work environment.	1.000	0.900	1.000
37	Original	*Debería existir la posibilidad de tener momentos de camaradería, intimidad y franqueza.* [There should be the possibility of having moments of comradery, intimacy, and frankness.]			
	Translated	There should be the possibility of having moments of harmonious coexistence with the team and openness.			
	Expert panel version	There should be the possibility of having moments of harmonious coexistence with the team and sincere openness.	1.000	0.900	1.000
40	Original	Debería existir una visión de familia del equipo de trabajo, una familia con conflictos, avenencias, pero unidos, ligados sentimentalmente. [There should be a feeling of family among the work team, a family with conflicts, agreements, but united, emotionally connected,]			
	Translated	There should be a feeling of family among the work team, as a family with conflicts, commitments, but united, emotionally connected.			
	Expert panel version	There should be a feeling of family among the work team despite conflicts and divergences.	1.000	0.800	1.000

* CVI—content validity index.

## Data Availability

The datasets generated and/or analyzed during the current study are not publicly available to preserve the anonymity of the respondents but are available from the corresponding author on reasonable request.
